# Psychometric testing of the Functional Assessment of Cancer Therapy/Gynecologic Oncology Group—Neurotoxicity (FACT/GOG-Ntx) subscale in a longitudinal study of cancer patients treated with chemotherapy

**DOI:** 10.1186/s12955-020-01493-y

**Published:** 2020-07-23

**Authors:** Hui Lin Cheng, Violeta Lopez, Simon Ching Lam, Anthony Kwun To Leung, Yu Chung Li, Kam Hung Wong, Joseph Siu Kie Au, Raghav Sundar, Alexandre Chan, Terrence Rong De Ng, Lorna Kwai Ping Suen, Choi Wan Chan, Janelle Yorke, Alex Molassiotis

**Affiliations:** 1 School of Nursing, The Hong Kong Polytechnic University, Hong Kong, Hong Kong SAR; 2 Alice Lee Centre for Nursing Studies, National University of Singapore, Singapore, Singapore; 3 Department of Clinical Oncology, Queen Elizabeth Hospital, Hong Kong, Hong Kong SAR; 4 Department of Oncology, Hong Kong Adventist Hospital, Hong Kong, Hong Kong SAR; 5 Department of Haematology-Oncology, National University Health System, Singapore, Singapore; 6 Department of Pharmacy, National University of Singapore, Singapore, Singapore; 7 Division of Nursing, Midwifery & Social Work, University of Manchester, Manchester, UK

**Keywords:** Chemotherapy, Peripheral neuropathy, FACT/GOG-Ntx, EORTC QLQ-CIPN20, Psychometrics, Responsiveness, Confirmatory factor analysis

## Abstract

**Background:**

The aim of this study was to evaluate the psychometric properties of the Functional Assessment of Cancer Therapy/Gynecologic Oncology Group—Neurotoxicity (FACT/GOG-Ntx) subscale in a longitudinal study of cancer patients treated with chemotherapy.

**Methods:**

Patients were assessed with the FACT/GOG-Ntx subscale, European Organization for Research and Treatment of Cancer Quality of Life Questionnaire Chemotherapy-Induced Peripheral Neuropathy Scale 20 (EORTC QLQ-CIPN20), National Cancer Institute -Common Terminology Criteria for Adverse Events (NCI-CTCAE), and light touch test using 10 g monofilament for up to ten assessment points from baseline (prior to initiation of first chemotherapy), after the end of each cycle (up to 6 cycles, 3 weeks per cycle), and at 6, 9, and 12 months after starting chemotherapy. Psychometric analyses included internal consistency reliability, convergent validity, factorial validity, sensitivity to change and responsiveness (minimal clinically important difference, MCID).

**Results:**

Cronbach’s alpha coefficients of the FACT/GOG-Ntx subscale were 0.82–0.89 across assessment points. The subscale strongly correlated with the EORTC QLQ-CIPN20 (*r* = 0.79–0.93) but low-to-moderately correlated with the NCI-CTCAE sensory (*r*_s_ = 0.23–0.45) and motor items (*r*_s_ = 0.15–0.50) as well as the monofilament test (*r*_s_ = 0.23–0.47). The hypothesized 4-factor structure of the FACT/GOG-Ntx subscale was not confirmed at assessment points (χ2/df = 2.26–8.50; all *P* < 0.001). The subscale exhibited small-to-moderate sensitivity to change (*r* = 0.17–0.37). The MCIDs were between 1.38 and 3.68.

**Conclusion:**

The FACT/GOG-Ntx subscale has satisfactory reliability, validity, sensitivity to change and responsiveness to evaluate CIPN in cancer patients. Future research is needed to explore the factorial structure of the FACT/GOG-Ntx subscale as the published four-factor structure was not supported in this study.

## Background

Chemotherapy-induced peripheral neuropathy (CIPN) is one of the most common neurological symptoms in patients treated with taxane- and/or platinum-based chemotherapy [1, 2]. The clinical presentation of CIPN across these two classes of chemotherapeutic agents are similar and predominantly sensory [3]. The consequences of CIPN are multifaceted in nature; including interference with daily life functioning and quality of life, psychological distress, and restrained socialization [4–7]. When CIPN symptoms become intolerable, chemotherapy dose deductions and delays may occur, as currently there are no proven effective treatments to manage CIPN [8]. The lack of an optimal method for assessing CIPN is suggested as a key barrier to impede effective symptom management [8].

Multiple methods exist to assess CIPN, encompassing objective tests, clinician-rated scales, and patient reported outcome *(PRO)* measures [9, 10]. Among these methods, *PRO* measures are unique in capturing the patients’ perspectives of CIPN concerns. In a systematic review of existing CIPN *PRO* measures, the Functional Assessment of Cancer Therapy/Gynecologic Oncology Group—Neurotoxicity (FACT/GOG-Ntx) subscale holds promise as a psychometrically sound measure [11]. The FACT/GOG-Ntx subscale, originally developed by the FACIT organization in collaboration with the Gynecologic Oncology group, is an 11-item neurotoxicity module added to the core quality of life measure of the FACT-G questionnaire [12]. The subscale as a standalone scale to assess CIPN has been previously validated in two relatively small samples (*n* = 56 and *n* = 134, respectively) of predominantly white gynecological cancer patients in the United States, and its preliminary cross-sectional reliability and validity is established [12, 13]. However, no study has tested the factorial validity of the FACT/GOG-Ntx subscale. To our knowledge, longitudinal validation of this subscale has not been performed in a large sample of patients with different cancer diagnoses.

An appropriate symptom assessment tool should have adequate sensitivity to change and responsiveness when used for assessing a change over time [14–16]. Sensitivity to change is defined as a measure’s ability to detect change but this is not sufficient to determine if the change is clinically meaningful [14]. Responsiveness refers to the ability of a measure to detect a clinically important change [16]. There are no published study examining the sensitivity to change and responsiveness of the FACT/GOG-Ntx subscale. Despite the FACT/GOG-Ntx subscale being utilized for almost 20 years, the psychometric data is still incomplete, particularly in longitudinal studies, thus its widespread use will be limited. This study aimed to evaluate the psychometric properties of the FACT/GOG-Ntx subscale in a longitudinal study of cancer patients treated with chemotherapy.

## Methods

### Study design

This study was conducted in Hong Kong, Singapore, and United Kingdom as part of a prospective longitudinal observational study of CIPN.

### Patients and settings

Patients were recruited from oncology departments of three hospitals in three countries/regions. Inclusion criteria were a) adult cancer patients (+ 18 years), b) chemotherapy-naive, c) about to receive taxane- and/or platinum-based chemotherapy, d) with expected survival of at least 12 months (as judged by the clinicians), and e) being able to provide written consent. Using convenience sampling, eligible patients were identified by research staff at each study site and approached for study briefing. Recruited patients provided written informed consent. The study was approved by the ethical review committee of each participating institution.

### Instruments

#### FACT/GOG-Ntx subscale

This scale is an 11-item *PRO* measure designed to capture CIPN symptoms [12]. Each item is scored on a 5-point scale (0 = not at all, 4 = very much), with a higher score reflecting worse CIPN. The English version was used for the UK and Singapore samples [13], with the traditional Chinese version for the Hong Kong sample [17]. The translated version was obtained from the FACIT organization which is the developer of the instrument. The FACIT organization has a standardized instrument translation process with seven steps, including forward translation from English to the target language by two bilingual speakers, back translation of reconciled version from the target language to English by one bilingual translator, independent reviews, and pilot test of the translated questionnaire with patients in the target language.

#### European Organization for Research and Treatment of Cancer quality of life questionnaire chemotherapy-induced peripheral neuropathy scale 20 (EORTC QLQ-CIPN20)

This *PRO* measure has 20 items assessing sensory, motor and autonomic aspects of CIPN. Items are rated from 1(not at all) to 4 (very much) and summed to produce a total score which is then linearly converted into a 0 to 100 scale. A higher score indicates worse CIPN. The psychometric properties of the EORTC QLQ-CIPN20 are established except for factorial validity [9, 18, 19]. Both English and Chinese versions of the questionnaires were used [17, 20].

#### National Cancer Institute -common terminology criteria for adverse events (NCI-CTCAE)

The NCI-CTCAE is a clinician-based scale to rate the frequency and severity of CIPN. It comprises two items: sensory- and motor neuropathy, with each item scored from 1 (asymptomatic) to 5 (death) [21]. Although the inter-rater reliability of the NCI-CTCAE without training is considered as suboptimal, it is found to be high (weighted *K* Cohen coefficients > 0.7) after training [22]. Training for the research staff at each site was provided to ensure consistency and accuracy in CIPN grading.

#### Light touch using standardized 10 g monofilament

This examination was applied to detect sensory impairment related to CIPN. The moderate-to-high agreements in and between examiners for monofilament tests are previously reported in cancer patients with CIPN (weighted *K* Cohen coefficients > 0.6) [22]. For this study, the research staff were trained to choose at least five testing sites of each finger/foot for the examination from the distal area to the proximal area. The result is binary, either normal or abnormal (defined as at least > 1 testing site with sensory problems).

### Data collection

Recruited patients were approached for data collection when they attended regular follow-up at the study sites. They were evaluated with the aforementioned instruments from baseline (prior to initiation of first chemotherapy), after the end of each cycle (up to 6 cycles, 3 weeks per cycle), and at 6, 9, and 12 months after starting chemotherapy.

### Psychometric testing

#### Floor/ceiling effects

The presence of floor/ceiling effects is determined by calculating the proportions of patients with the lowest/highest scores at the scale level. The most commonly used cut-off point of 15% was adopted as the criterion [23].

#### Internal consistency reliability

Cronbach’s alpha coefficients were calculated to evaluate internal consistency reliability of the FACT/GOG-Ntx subscale, with values greater than 0.7 being viewed as adequate [23]. Corrected item-total correlations and item-item correlations were also supplemented to reflect the scale’s homogeneity, and a correlation coefficient value falling between 0.3 and 0.7 is recommended [24].

#### Convergent validity

Pearson’s production-moment correlation coefficients were employed to measure the strength of linear relationships between the FACTT/GOG-Ntx subscale with EORTC QLQ-CIPN20 questionnaire. Spearman’s rank correlation coefficients were used to examine the associations of FACT/GOG-Ntx subscale with NCI-CTCAE scales and light touch test because the latter variables were ordinal. A correlation coefficient greater than 0.4 is an indicator of convergent validity [25].

#### Factorial validity

As informed by Huang et al’s validation study (although detailed results are not reported), the FACT/GOG-Ntx subscale is hypothesized with four factors, encompassing sensory (4 items), motor (3 items), hearing (2 items) and dysfunction (2 items) [13]. Confirmatory factor analyses (CFA) with maximum likelihood estimation method were used to test the goodness-of-fit of the four-factor structure of the FACT/GOG-Ntx subscale at each assessment point [26]. Several model fit indices and related criteria were adopted, including Chi-square/df ratio (χ^2^/df; < 3 acceptable), Comparative Fit Index (CFI; > 0.95 acceptable), Tucker-Lewis Index (TLI; > 0.95 acceptable), Root Mean Square Error of Approximation (RMSEA; < 0.08 acceptable), and Standardized Root Mean Square Residual (SRMR,< 0.05 acceptable) [27].

#### Sensitivity to change

Although a variety of statistical methods have been used to assess sensitivity to change of a measure, no single one is superior to the other [14]. For this study, sensitivity to change was evaluated by examining changes in scores of the FACT/GOG-Ntx subscale over time using two methods. Firstly, generalized estimating equation was used as there was non-normally distributed data and missing data at specific assessment points (mostly due to completion of or changing chemotherapy) [28]. Secondly, to allow a comparison with prior studies, effect size (ES) was calculated based on the formula $r=\frac{Z}{\sqrt{N}}$, where 0.1, 0.3 and 0.5 were considered as small-, moderate- and large-ES, respectively [29].

#### Responsiveness

Responsiveness of the FACT/GOG-Ntx subscale was assessed using minimal clinically important difference (MCID) estimates reflecting the smallest changes in an outcome that patients would perceive as beneficial [16]. Ideally, the MCID of a measure is determined using a combination of anchor-based and distribution-based approaches. As anchor-based methods are not feasible due to no well-established external criterion, only the distribution-based methods were used to estimate the MCID of the FACT/GOG-Ntx subscale. Yost and Eton [30] suggested that one-third and half standard deviations (SD) are the closest estimates for determining the MCID of the FACT specific subscales. Therefore, the MCID values were calculated using these two estimation methods, including 0.3 SD and 0.5 SD, where SD was applied to the baseline FACT/GOG-Ntx subscale score.

Data were entered and analyzed using IBM SPSS Statistics 23.0. For CFA, AMOS 22.0 was used. The statistically significant level was set at *p* < 0.05.

## Results

### Patient characteristics

Table 1 shows the characteristics of participants at baseline. Of 343 consented patients, 213 were recruited from Hong Kong, 94 from Singapore and 36 from the UK. Patients were 55. 2 years on average (SD = 9.40; range = 33–79). They were predominantly female (74.6%), Chinese (78.4%), and had cancer stage I-III (77.8%). Half of them were diagnosed with breast cancer (50.7%) and received adjuvant chemotherapy (58%). Taxane-based chemotherapy (45.2%) was the most commonly used chemotherapeutic protocol.

**Table 1:** Socio-demographic and clinical characteristics of participants (*N* = 343)

Characteristics	Mean	SD (Range)
Age	55.2	9.4 (33–79)
Gender	N	%
Male	87	25.4
Female	256	74.6
Race		
Chinese	269	78.4
Non-Chinese Asian	31	9.0
Caucasian	43	12.5
Cancer diagnosis		
Breast	174	50.7
Lung	48	14.0
Gynecological	45	13.1
Head & Neck	30	8.7
Gastrointestinal	29	8.5
Urinary tract	17	5.0
Cancer stage		
I	52	15.2
II	99	28.9
III	116	33.8
IV	76	22.2
Treatment intent		
Adjuvant	199	58.0
Neo-adjuvant	51	14.9
Concurrent	30	8.7
Palliative	63	18.4
Type of chemotherapy		
Taxanes	155	45.2
Platinum	109	31.8
Combination	79	23.0

For this study, sample size ranged from 118 to 340 from baseline to the 12-month follow-up. Data at each assessment point were missing for many reasons, including completion of the pre-specified chemotherapy protocol, discontinuation of chemotherapy due to medical reasons, or not willing to complete the questionnaire due to physical and psychological adverse effects of chemotherapy, or death.

### Psychometric properties

Table 2 presents the results of psychometric testing.

**Table 2:** Psychometric properties of the FACT/GOG-Ntx subscale^e^ over time

Properties	Methods	Analytical methods and criteria	Results in this study	Results in prior studies
Floor/ceiling effect	Examining the proportions of participants with the lowest/highest scores at the scale level	Frequency endorsement (15% of endorsement with lowest/highest scores)	Floor effects (28.3–50.6%)	NA
Internal consistency	Cronbach’s method	Cronbach’s alpha statistics (alpha > 0.70)	Total score: 0.82–0.89 Sensory^d^: 0.80–0.90 Motor^d^: 0.70–0.79 Hearing^d^: 0.64–0.90 Dysfunction^d^: 0.71–0.96	Total score only: 0.82–0.86^a^ 0.62–0.90^b^ 0.80–0.85^c^
	Examining the corrected item-total correlations	Pearson product-moment correlation coefficient (*r* = 0.30–0.70)	Q1 (Numbness or tingling in hands): 0.47–0.71 Q2 (Numbness or tingling in feet): 0.57–0.73 Q3 (discomfort in hands): 0.59–0.73 Q4 (discomfort in feet): 0.66–0.76 Q5 (joint pain or muscle cramps): 0.44–0.64 Q6 (weak all over): 0.47–0.72 Q7 (trouble hearing): 0.30–0.59 Q8 (ringing or buzzing in ears): 0.26–0.51 Q9 (trouble buttoning buttons): 0.34–0.63 Q10 (trouble feeling the shape of small objects): 0.34–0.65 Q11 (trouble walking): 0.55–0.69	Q1: 0.37–0.69^c^ Q2: 0.37–0.81^c^ Q3: 0.30–0.73^c^ Q4: 0.35–0.78^c^ Q5: 0.15–0.44^c^ Q6: 0.39–0.58^c^ Q7: 0.19–0.27^c^ Q8: 0.13–0.38^c^ Q9: 0.21–0.46^c^ Q10: 0.24–0.48^c^ Q11: 0.35–0.52^c^
Convergent validity	Examining the correlation between the FACT/GOG-Ntx and the other instruments with similar construct	Spearman’s correlation coefficient (r_s_ > 0.40)	EORTC QLQ-CIPN20^e^: 0.79–0.93 (p < 0.01) NCI-CTCAE (sensory)^e^: 0.23–0.45 (p < 0.01) NCI-CTCAE (motor)^e^: 0.15–0.50 (*p* < 0.01) Light touch using 10-g Monofilament^e^: 0.23–0.47 (p < 0.01)	NA
Factorial validity	Examining the hypothesized factor structure of the scale	Confirmatory factor analysis (χ^2^/df < 3, CFI > 0.95, TLI > 0.95, RMSES < 0.08)	χ^2^/df = 2.26–8.50, CFI = 0.79–0.95, TLI = 0.63–0.91, RMSEA = 0.07–0.14; SRMR = 0.046–0.079,	NA
Sensitivity to change	Measuring changes in scores of the FACT/GOG-Ntx subscale over time	Generalized estimating equation (*p* < 0.05)	Wald chi-square = 113.6, p < 0.001	N/A
		Effect size $r=\frac{Z}{\sqrt{N}}$ (*r =* 0.1, 0.3 and 0.5 as small-, moderate- and large effect size)	*r =* 0.17–0.37	*r =* 0.37–0.91^a^
Responsiveness	Examining the MCID of the FACT/GOG-Ntx subscale	0.3 SD and 0.5 SD of the baseline FACT/GOG-Ntx subscale score.	MCID = 1.38–3.68	N/A

Note: FACT/GOG-Ntx subscale = Functional Assessment of Cancer Therapy/Gynecologic Oncology Group—Neurotoxicity subscale; EORTC QLQ-CIPN20 = European Organization for Research and Treatment of Cancer Quality of Life-Chemotherapy-Induced Peripheral Neuropathy Scale 20; NCI-CTCAE = National Cancer Institute -Common Terminology Criteria for Adverse Events; CFI = comparative fit index; TLI = Tucker-Lewis index; RMSEA = root mean square error of approximation; SRMR = standardized root mean square residual; MCID = minimal clinically important difference; SD = standard deviation

^a^Cella et al. (2003) [31]: 230 patients with advanced non-small cell lung carcinoma

^b^Calhoun et al.(2003) [12]: 56 chemotherapy-naive ovarian patients

^c^Huang et al. (2007) [13]: 134 advanced endometrial cancer patients

^d^The hypothesized four-factor structure of the FACT/GOG-Ntx subscale was proposed by Huang et al. (2007) and not confirmed in this study

^e^A higher score indicates worse neurotoxicity

#### Floor/ceiling effects

Of all patients, 28.3–50.6% and 0.3–0.8% rated the lowest- and highest score on FACT/GOG-Ntx subscale, respectively.

#### Internal consistency reliability

Cronbach’s alpha coefficients of the FACT/GOG-Ntx subscale were 0.82–0.89 from baseline to 12-month follow-up. Further investigation of each domain found that Cronbach’s alpha coefficients were stably adequate for three of the four domains except for the hearing domain (0.64–0.90) at different time points. Corrected item-total correlations for all scale items were adequate except for item 4 (discomfort in the feet) slightly exceeding 0.70 (*r* = 0.66–0.79) at 8 of 10 assessment points. The item-item correlation coefficients for item 7 (trouble hearing) and item 8 (ringing or buzzing in ears) related to hearing dysfunction were slightly less than 0.30 across different time points. However, removal of these items did not result in significant changes in Cronbach’s alpha coefficients (≥0.1).

#### Convergent validity

The FACT/GOG-Ntx subscale scores showed moderate-to-high associations with the EORTC QLQ-CIPN20 scores at all assessment points (*r* = 0.79–0.93, *p* < 0.01). Significant but low-to-moderate correlations were found between the FACT/GOG-Ntx subscale and the NCI-CTCAE sensory/motor item over time (sensory *r*_s_ = 0.23–0.45; motor *r*_s_ = 0.15–0.50, all *p* < 0.01). Correlations between the two measures were lower at baseline and gradually increased, reaching and maintaining *r*_s_ = 0.40 and higher around the end of chemotherapy until the 12-month follow-up. A similar correlation pattern was noted between FACT/GOG-Ntx subscale and light touch test at all the assessment points except for baseline (*r*_s_ = 0.23–0.47). (Table 3).

**Table 3:** Correlations between FACT/GOG-Ntx subscale scores and other measures’ scores at each assessment point

Scales	Ntx subscale^a^									
	Baseline (n = 343)	Cycle 1 (*n* = 307)	Cycle 2 (*n* = 286)	Cycle 3 (*n* = 270)	Cycle 4 (*n* = 240)	Cycle 5 (*n* = 138)	Cycle 6 (*n* = 118)	6mFU (*n* = 254)	9mFU (*n* = 235)	12mFU (*n* = 195)
EORTC QLQ CIPN-20	0.852**	0.849**	0.891**	0.813**	0.890**	0.897**	0.902**	0.925**	0.925**	0.794**
NCI-CTCAE ^3^										
Motor	0.145**	0.264**	0.301**	0.342**	0.391**	0.488**	0.496**	0.439**	0.435**	0.467**
Sensory	0.10	0.231**	0.340**	0.357**	0.370**	0.414**	0.376**	0.428**	0.446**	0.428**
Light touch using 10-g Monofilament	0.078	0.234**	0.249**	0.226**	0.257**	0.452**	0.280**	0.447**	0.473**	0.277**

Note: FACT/GOG-Ntx subscale = Functional Assessment of Cancer Therapy/Gynecologic Oncology Group—Neurotoxicity subscale; EORTC QLQ-CIPN20 = European Organization for Research and Treatment of Cancer Quality of Life-Chemotherapy-Induced Peripheral Neuropathy Scale 20; NCI-CTCAE = National Cancer Institute -Common Terminology Criteria for Adverse Event; m=month; FU= follow-up

** p < 0.01

^a^Calculated means for 0.3SD and 05 SD for the subscale at baseline and follow-up time points

#### Factorial validity

The CFA indicated that the fit indexes did not meet the standard of all fit criteria [26] at each assessment point: Chi Square/df ratio = 2.26–8.50 (*p* < 0.001), CFI = 0.79–0.95, TLI = 0.63–0.91, RMSEA = 0.07–0.14, SRMR = 0.046–0.079, indicating that the hypothesized four-factor model proposed by Huang et al. [13] did not satisfactorily fit the current sample over time.

#### Sensitivity to change

The estimated marginal means of the FACT/GOG-Ntx subscale scores significantly decreased over time (Wald chi-square = 113.6, *p* < 0.001). Post-hoc comparison analysis indicated that the estimated marginal means of the FACT/GOG-Ntx subscale scores significantly decreased from 42.0 at baseline to 40.7 at cycle 1 (p < 0.001), remained decreased until cycle 6, but significantly increased thereafter from 38.8 at 6-month follow-up to 39.7 at 12-month follow-up (*p* < 0.05). The magnitude of ES was small at the first two cycles (*r* = 0.17–0.25), but significantly increased with the number of chemotherapy cycles, until peaking at 6 month follow-up (*r* = 0.37), decreasing thereafter.

#### Responsiveness

The distribution-based method yielded MCID values of 1.38 to 2.21 using a 0.3SD (baseline to each assessment point change) and 2.30 to 3.68 using 0.5 SD (Table 4).

**Table 4:** Distribution-based approach to estimate the MCID ^1^ of the FACT-GOG-Ntx Subscale at different time points

FACT/GOG-Ntx subscale	0.3SD	0.5SD
Baseline (T1)	1.07	1.79
Cycle 1(T2)	1.52	2.53
Cycle 2(T3)	1.73	2.88
Cycle 3(T4)	1.95	3.26
Cycle 4(T5)	1.73	2.89
Cycle 5(T6)	2.06	3.43
Cycle 6 (T7)	1.97	3.28
6 month follow-up (T8)	1.88	3.14
9 month follow-up (T9)	1.85	3.09
12 month follow-up (T10)	1.49	2.48
Change from T1 to T2	1.54	2.57
Change from T1 to T3	1.77	2.95
Change from T1 to T4	1.94	3.23
Change from T1 to T5	1.77	2.95
Change from T1 to T6	2.21	3.68
Change from T1 to T7	1.91	3.18
Change from T1 to T8	1.71	2.85
Change from T1 to T9	1.61	2.69
Change from T1 to T10	1.38	2.30

*MCID* minimal clinically important difference; *SD* standard deviation;

## Discussion

Consistent with previous validation studies, Cronbach’s alpha coefficients of the FACT/GOG-Ntx subscale were above 0.8 from baseline to 12-month follow-up [13, 31]. Item analysis found that almost all of the 11 items had adequate corrected item-total correlations except for item 4 (discomfort in the feet) slightly exceeding 0.7 at most assessment points, suggesting item redundancy. The wording “discomfort” in this item is ambiguous and can be broad enough to also indicate numbness, tingling, or muscle weakness problems as measured by other items. Thus, this item may be overlapping with other similar items in the scale and further revision of it can be considered. Furthermore, item analysis also found that item 7 and item 8 had slightly low to moderate item-item correlations, suggesting that hearing dysfunction may be less relevant to CIPN or may represent a preexisting condition unrelated to CIPN. The results may be due to the fact that patients endorsed less concerns related to hearing problems. Past research shows that the occurrence of hearing dysfunction is more common in patients receiving cisplatin [32]. As only a small proportion of patients (12.2%) were on cisplatin therapy in the present study, this may explain why hearing problems might be less common.

As expected, convergent validity of the FACT/GOG-Ntx subscale was established by its strong relationship with EORTC QLQ-CIPN20 because both PRO measures assess the similar CIPN symptoms. By contrast, low-to-moderate correlations between FACT/GOG-Ntx and NCI-CTCAE were observed, highlighting inconsistency in CIPN evaluation between clinician-based scales and PRO measures, which is in accordance with prior findings in terms of poor correlations between NCI-CTCAE and other CIPN PRO measures including with the EORTC QLQ-CIPN20 and the Patient Neurotoxicity Questionnaire [22, 33]. Similar low-to-moderate correlations were noted between monofilament examination and FACT/GOG-Ntx subscale too, which echoes previous findings [34]. This can be explained by the scope of two different tests. The monofilament test aims to diagnose sensory loss (pathological) as an early signal of CIPN, while FACT/GOG-Ntx subscale relies on patient self-reports of CIPN during the past week and extends beyond sensory problems including motor, functional and other autonomic problems.

The 11-item FACT/GOG-Ntx subscale was hypothesized as a multidimensional scale with four factors at the time of development and validation [11], but its factor structure has not been confirmed particularly in a large sample of mixed cancer patients [12, 13, 17]. In this study, the four-factor model of the FACT/GOG-Ntx proposed by Huang et al. [13] was not verified using CFA based on model fit indices and criteria. If the factor structure of the scale is not confirmed, exploratory factor analysis should be conducted in the next step of the research. Our exploratory analysis of data at 6-month follow-up (data not shown in this manuscript) identified a three-factor structure after item 5 and item 11 were deleted. Of the three factors, two factors pertained to sensory and motor problems in the upper extremities; and the other one related to hearing problems and body weakness. However, this 3-factor structure of the FACT/GOG-Ntx subscale was not supported at the other assessment points. Thus, further exploration of the factor structure of the FACT/GOG-Ntx scale in a separate sample is warranted.

Sensitivity to change of the FACT/GOG-Ntx subscale was also confirmed in this study. This result is in accordance with a previous validation study of lung cancer patients, although the latter study reports a moderate to high ES (r = 0.37–0.91) [31]. The difference in ES across these two studies might be related to different patient profiles as the previous study included advanced cancer patients requiring more aggressive chemotherapy.

In this study, the MCID of the FACT/GOG-Ntx subscale were 1.38 to 3.68 across the ten assessment points. These estimates were lower than the defined MCIDs of 3.3 to 4.4 point change for the 11-item FACT/GOG-Ntxt based upon Yost and Eton (2005)‘s recommendation of 0.3–0.4 point change per item for FACT-specific subscale [30]. As the MCID of a patient-reported outcome measure is dependent on the context, the distribution-based methods allow researchers to have more accurate precisions of measurements, thus our results may reflect the responsiveness of the FACT/GOG-Ntx subscale. For patients with CIPN, even with small changes in scores, healthcare providers should be aware of possible deteriorations in CIPN-related concerns.

Despite an advantage of a large sample with different ethnic groups, this study has a few limitations. The FACT/GOG-Ntx subscale exhibited floor effects over time, along with a large proportion of missing data (mostly due to completion of or changing chemotherapy) at some assessments points, which may bias the results. Notably, this paper is a secondary analysis of the psychometric properties of the FACT/GOG-Ntx used in the main study. The sample size was not planned for this paper but was estimated based on the primary aim of the main study investigating the progression and risk factors of CIPN in cancer patients. However, the current sample size was 343, which far exceeded the minimal sample size of 200 required to achieve accurate inferences in the CFA [26]. Furthermore, the translation as well as face and content validity of the traditional Chinese version of the FACT/GOG-Ntx were conducted by one researcher only in Taiwan, where the Chinese language used has some differences from the Hong Kong dialect. Given the possible socio-cultural influences on the wordings and expressions of certain items, face and content validity testing of the scale should be performed in the context of Hong Kong. Lastly, we recruited patients who have received taxane- and platinum-based chemotherapy, the subscale’s psychometric properties in patients receiving other chemotherapy drugs or with longer-term CIPN cannot be ascertained. We recommend to further investigate the construct validity of the FACT/GOG-Ntx subscale in cancer patients receiving other types of chemotherapy or those affected by long-term CIPN and to expand its applicability across different cancer populations.

## Conclusions

This study demonstrated the FACT/GOG-Ntx subscale has satisfactory reliability, validity, sensitivity to change and responsiveness to evaluate CIPN in cancer patients. Future research is needed to explore the factorial structure of the FACT/GOG-Ntx subscale as the published four-factor structure was not supported in this study.

## Data Availability

The datasets used and/or analyzed during the current study are available from the corresponding author on reasonable request.

## Abbreviations

FACT/GOG-Ntx subscale: Functional Assessment of Cancer Therapy/Gynecologic Oncology Group—Neurotoxicity subscale
EORTC QLQ-CIPN20: European Organization for Research and Treatment of Cancer Quality of Life-Chemotherapy-Induced Peripheral Neuropathy Scale 20
NCI-CTCAE: National Cancer Institute -Common Terminology Criteria for Adverse Events
CFI: Comparative Fit Index
TLI: Tucker-Lewis index
RMSEA: Root Mean Square Error of Approximation
SRMR: Standardized Root Mean Square Residual
MCID: Minimal Clinically Important Difference
SD: Standard Deviation
CIPN: Chemotherapy-induced Peripheral Neuropathy
PRO: Patient-reported Outcome
ES: Effect Size
CFA: Confirmatory Factor Analysis
